# DNA barcoding does not separate South American *Triatoma* (Hemiptera: Reduviidae), Chagas Disease vectors

**DOI:** 10.1186/s13071-014-0519-1

**Published:** 2014-11-21

**Authors:** Silvia Andrade Justi, Carolina Dale, Cleber Galvão

**Affiliations:** Universidade Federal do Rio de Janeiro, Instituto de Biologia, Laboratório de Biologia Evolutiva Teórica e Aplicada, Rio de Janeiro, Brazil; Laboratório Nacional e Internacional de Referência em Taxonomia de Triatomíneos, InstitutoOswaldo Cruz, FIOCRUZ, Rio de Janeiro, Brazil

**Keywords:** Triatominae, Chagas disease, DNA barcoding, Molecular identification

## Abstract

**Background:**

DNA barcoding assumes that a biological entity is completely separated from its closest relatives by a *barcoding gap*, which means that intraspecific genetic distance (from COI sequences) should never be greater than interspecific distances. We investigated the applicability of this strategy in identifying species of the genus *Triatoma* from South America*.*

**Findings:**

We calculated intra and interspecific Kimura-2-parameter distances between species from the *infestans*, *matogrossensis*, *sordida* and *rubrovaria *subcomplexes. In every subcomplex examined we observed at least one intraspecific distance greater than interspecific distances.

**Conclusions:**

Although DNA barcoding is a straightforward approach, it was not applicable for identifying Southern American *Triatoma* species*,* which may have diverged recently. Thus, caution should be taken in identifying vector species using this approach, especially in groups where accurate identification of taxa is fundamentally linked to public health issues.

## Findings

DNA barcoding, as proposed by Hebert et al. [1] assumes that a biological entity is completely separated from its closest relatives by a *barcoding gap* [2], which means that intraspecific genetic distances (from COI sequences) are never greater than interspecific distances.

*Triatoma *Laporte (Hemiptera: Reduviidae) is the most diverse genus of Chagas Disease vectors, and accurate identification of species is imperative for the efficiency of vector control programs. The *Triatoma* genus is divided into species complexes and subcomplexes according to geographic distribution and morphological similarity [3].

Recently, Justi et al. [4] reported that the relationships between species assigned to South American *Triatoma*subcomplexes could not be untangled with the data in hand. We were then prompted to investigate whether DNA barcoding would be a useful tool for identifying the species within the *infestans*, *matogrossensis*, *sordida* and *rubrovaria *subcomplexes [3].

Kimura-2-parameter genetic distances [5] were calculated pairwise within each of the above mentionedsubcomplexes (Table 1) using the software MEGA v. 5 [6], and intra and interspecific distances were compared.

**Table 1:** **K2p-distances between species of the** ***Triatoma*** **subcomplexes studied**

**Subcomplex**	**GenBank**	**Number**	**Geographic Origin**												
*infestans*					1	2	3	4	5						
	KC249330	1	Chaco Tita, Cochabamba, Bolivia	*T. delpontei* 53											
	KC249346	2	Chaco Tita, Cochabamba, Bolivia	*T. infestans* 44	***0.021***										
	KC249349	3	Cotapachi, Cochabamba, Bolivia	*T. infestans* 58	***0.025***	0.018									
	KC249352	4	Mataral, Cochabamba, Bolivia	*T. infestans* 60	***0.025***	0.018	0.005								
	KC249354	5	Ilicuni, Cochabamba, Bolivia	*T. infestans* 63	***0.021***	0.016	0.000	0.006							
	KC249355	6	Montevideo, Uruguai	*T. infestans* 69	0.072	***0.061***	***0.064***	***0.069***	***0.103***						
*matogrossensis*					7	8	9	10	11	12					
	KC249327,KC249328	7	Posse, GO, Brazil	*T. costalimai* 35											
	KC249329	8	Chiquitania, Cochabamba, Bolivia	*T. costalimai* 42	0.154										
	KC249360	9	São Gabriel D'oeste, MS, Brazil	*T. matogrossensis* 192	0.134	0.138									
	KC249361	10	Bahia, Brazil	*T. matogrossensis* 31	0.151	0.152	***0.047***								
	KC249391	11	Pantanal, MT, Brazil	*T. vandae* 28	0.156	0.151	0.047	0.040							
	KC249392	12	Rio Verde do MatoGrosso, MT, Brazil	*T. vandae* 73	0.138	0.146	***0.005***	0.046	0.045						
	KC249393,KC249394	13	Rondonópolis, MT, Brazil	*T. vandae* 74	0.158	0.150	0.048	0.059	0.007	0.052					
*rubrovaria*					14	15	16	17	18	19	20	21	22	23	24
	KC249322	14	São Gerônimo, RS, Brazil	*T. carcavalloi* 78											
	KC249323	15	Caçapava do Sul, RS, Brazil	*T. circummaculata* 120	0.039										
	KC249324	16	Sítio Faxina, Piratini, RS, Brazil	*T. circummaculata* 121	0.029	0.025									
	KC249325	17	Sítio Faxina, Piratini, RS, Brazil	*T. circummaculata* 122	0.017	***0.039***	***0.033***								
	KC249356	18	Nova Petrópolis, RS, Brazil	*T. klugi* 75	0.018	***0.037***	***0.031***	***0.017***							
	KC249369	19	Sítio Faxina, Piratini, RS, Brazil	*T.rubrovaria* 123	***0.055***	0.023	0.029	0.055	0.057						
	KC249370	20	Sítio venda da Lagoa, Canguçu, RS, Brazil	*T.rubrovaria* 134	0.065	0.052	0.065	0.065	0.061	0.070					
	KC249372	21	SítioFaxina, Pinheiro Machado, RS, Brazil	*T.rubrovaria* 136	0.042	0.019	0.027	0.036	0.036	0.031	0.035				
	KC249373	22	Sítiovenda da Lagoa, Canguçu, RS, Brazil	*T.rubrovaria* 140	0.038	0.020	0.019	0.043	0.040	0.029	0.032	0.012			
	KC249374	23	Canguçu, RS, Brazil	*T.rubrovaria* 156	0.039	0.020	0.019	0.045	0.042	0.029	0.032	0.012	0.000		
	KC249375	24	Caçapava do Sul, RS, Brazil	*T.rubrovaria* 76	0.021	0.029	0.021	0.016	0.016	0.033	***0.074***	0.034	0.038	0.038	
	KC249376	25	Quevedos, RS, Brazil	*T.rubrovaria* 77	0.029	0.030	0.043	0.022	0.029	0.046	***0.065***	0.031	0.046	0.048	0.026
*sordida*					26	27	28	29	30	31	32	33	34		
	KC249338	26	Rivadaria, Argentina	*T. garciabesi* 89											
	KC249342	27	Santa Cruz, Bolívia	*T. guasayana* 55	0.077										
	KC249343	28	Santa Cruz, Bolívia	*T. guasayana* 82	0.065	0.056									
	KC249379,KC249380	29	Romerillo, Cochabamba, Bolivia	*T. sordida* 46	0.029	0.060	0.060								
	KC249381,KC249382	30	Romerillo, Cochabamba, Bolivia	*T. sordida* 47	***0.030***	0.061	0.061	0.000							
	KC249383	31	La Paz, Bolívia	*T. sordida* 83	0.081	0.013	0.063	0.066	0.066						
	KC249384	32	Pantanal, MS, Brazil	*T. sordid*a 85	0.069	0.012	0.062	0.065	***0.065***	0.025					
	KC249385	33	Santa Cruz, Bolívia	*T. sordida* 86	0.043	0.082	0.074	0.035	***0.035***	0.073	0.082				
	KC249387	34	San Miguel Corrientes, Argentina	*T. sordida* 88	0.061	0.058	0.063	0.070	***0.071***	0.058	0.055	0.052			
	KC249388	35	Poconé, MT, Brazil	*T. sordida* 90	0.069	0.017	0.058	0.075	***0.075***	0.031	0.011	0.078	0.051		

Highlighted distances deviate from the DNA barcoding premis that intraspecific distances are smaller than interspecific distances.

In all subcomplexes we observed at least one intraspecific distance greater than interspecific distances (Table 1). To be considered appropriate to identify species within a group, intraspecific distances must always be greater than interspecific ones [2], and therefore DNA barcoding is not accurate for the species-level identification of South American *Triatoma*. Moreover, the method fails to account for hybridization events, which are naturally observed in *Triatoma* [7,8], and introgression, which is frequent in nuclear DNA [9]. These considerations argue that Hebert *et al.*’s [1] proposal of cataloguing biodiversity based only on DNA barcoding may severely underestimate it.

Besides that, as highlighted by Dujardin *et al.* [10], the morphological changes observed in closely related “species”, or “lineages” as we prefer to call them, may have led taxonomists to rush into describing subspecies or species, even genera. Molecular phylogenetic studies are in their infancy in unravelling the evolution of Triatominae, and a comprehensive molecular phylogeny, including more than one specimen for most lineages, was published only in 2014 [4], although several analyses were conducted focusing on small species groups. Taken together, these statements make it clear that further investigations of Triatominae evolution are long overdue, preferably integrating morphological, molecular and ecological data.

Lineage evolution has not occurred, but it is happening now. Concerning lineages designated in the *infestans* complex (including the subcomplexes studied here), separation is much clearer in terms of morphology than in molecular systematics. In cases where lineages have not reached reciprocal monophyly, defining taxonomic entities is not a straightforward issue [11]. Therefore caution is necessary, especially in a group where accurate identification of taxa is fundamentally linked to public health issues.

## Conclusions

Although DNA barcoding is a straightforward approach, it was not applicable for identifying Southern American *Triatoma* species*,* which may have diverged recently. Thus, caution should be taken in identifying vector species using this approach, especially in groups where accurate identification of taxa is fundamentally linked to public health issues.
